# Clinical Pain and Neuropsychological Functioning in Parkinson's Disease: Are They Related?

**DOI:** 10.1155/2016/8675930

**Published:** 2016-01-13

**Authors:** Gwenda Engels, Wouter D. Weeda, Annemarie M. M. Vlaar, Henry C. Weinstein, Erik J. A. Scherder

**Affiliations:** ^1^Department of Clinical Neuropsychology, Faculty of Behavioral and Movement Sciences, VU University, Van Der Boechorststraat 1, 1081 BT Amsterdam, Netherlands; ^2^Department of Neurology, OLVG West, Jan Tooropstraat 164, 1061 AE Amsterdam, Netherlands; ^3^Department of Methodology and Statistics, Leiden University, Wassenaarseweg 52, 2333 AK Leiden, Netherlands

## Abstract

*Introduction.* Pain is an important nonmotor symptom of Parkinson's disease (PD). Brain areas such as the hippocampus and the prefrontal cortex play an important role in the processing of pain. Since these brain areas are also involved in cognitive functioning, for example, episodic memory and executive functions, respectively, we examined whether a relationship exists between cognitive functioning and spontaneous pain in PD.* Methods*. Forty-eight patients with PD and 57 controls participated. Cognitive functioning was measured by a comprehensive battery of neuropsychological tests. Both the sensory-discriminative aspect and the motivational-affective aspect of pain were assessed. Multiple linear regression analyses were performed to assess a relation between cognition and pain.* Results*. Cognition was related to neither the sensory nor the affective aspect of pain in our sample of PD patients. Variance in pain measures was primarily explained by symptoms of depression and anxiety.* Discussion*. The difference between the affective and the sensory aspect of pain might be due to the neuropathology of PD, which is mainly present in areas processing the affective aspect of pain. Pain treatment might improve when mood is taken into account. We provide several explanations for the lack of an association between pain and cognition.

## 1. Introduction

Nonmotor manifestations of Parkinson's disease (PD) have received increasingly more attention over the past few decades, which is justified considering its impact on everyday life of PD patients [1]. Pain is one of these nonmotor manifestations, which has a large impact on quality of life [2, 3]. Pain is estimated to be present in two-thirds of PD patients [4] and has been reported already at the clinical onset of the disease [5, 6]. Additionally, a difference between PD patients and controls has been found in sensitivity to pain: both the tolerance and threshold of pain appear lowered in PD [7]. Thus, painful stimuli might be more painful for PD patients than for controls. The importance for everyday clinical practice is further emphasized when the possibility of an undertreatment of pain in this group was suggested by a study from Beiske and colleagues, who found that only half of the PD patients with pain received analgesics or physiotherapy [8].

Pain is a complex psychological and neurophysiological phenomenon and is processed in a neural network, involving the lateral and the medial pain system [9]. The lateral pain system is mainly involved in processing sensory-discriminative aspect (e.g., localization, intensity) of pain. It consists of the spinothalamic tract, which passes through the lateral thalamus and projects mainly towards sensory cortical areas. The medial pain system mainly processes motivational-affective and cognitive-evaluative aspects of pain (e.g., unpleasantness, suffering) and projects through medial thalamic nuclei to cognition- and emotion-related areas, for example, the hippocampus and anterior cingulate cortex (ACC). For a detailed overview of these pain systems, the reader is referred elsewhere [10–14]. The medial pain system appears to be primarily affected by the neuropathology of PD [15].

Pain in PD has been linked to various factors, such as dopaminergic fluctuations [16, 17], depressive symptoms [18], duration of disease [7], motor problems [19], and cognitive functioning [15]. Impairment of cognitive functioning, in particular, executive functioning (EF) and memory, is common in PD patients [20, 21]. In the general population, the relation between cognition and pain has been assessed before. Cognitive functions, such as executive functioning and attention, are thought to be inversely related to pain, implying that less pain coincides with better cognitive functioning. Performance on a cognitive test was related to acutely induced experimental pain in a study of Seminowicz and Davis [22]. Processing of pain was found to show significant overlap with an attention-specific network, taking away resources for cognitive performance: pain demands attention [22]. More complex relations have been suggested as well. For example, the degree to which cognitive performance is affected might depend on the difficulty of the task and the intensity of pain [23–25]. In a study from Pickering and colleagues it was shown that the interaction between cognition and pain is not restricted to a* concurrent* cognitive task: a higher score on the Mini-Mental State Examination (MMSE, measured off-line) correlated with a higher pain tolerance (assessed by psychophysical mechanical and thermal techniques) in healthy elderly. The authors explain this relation by the ability to centrally integrate the multiple facets of pain [26]. The fact that there is a convincing overlap in areas involved in pain processing and areas involved in cognitive performance emphasizes the association between these functions.

Many of the pain processing areas eventually become affected in PD [27, 28]. Brainstem areas involved in suppression of pain, such as the periaqueductal grey and locus coeruleus, are affected already in an early stage of the disease. This might result in an increase of pain in PD patients, but when neocortical areas are affected in later stages of the disease, pain might decrease [15]. In other words, pain might subside when cognitive impairment progresses in PD. However, if a higher level of pain does remain and cognitive impairment worsens, patients might not be able to communicate their pain. This would hinder successful treatment. Therefore, the goal of the present study is to examine the possible relationship between cognitive functioning and pain in PD patients.

## 2. Methods

### 2.1. Participants

#### 2.1.1. Inclusion Criteria

Patients were included when they had a diagnosis of PD according to the UK PD Society Brain Bank diagnostic criteria [29]. This diagnosis was confirmed by a neurologist. Furthermore, the subjects had no history of cerebral traumata or of psychiatric disorders unrelated to PD (e.g., schizophrenic episodes, major depressive disorder long before the onset of PD) and were able to understand the neuropsychological tests (i.e., MMSE score > 16).

#### 2.1.2. Subject Characteristics

Forty-eight PD patients and 57 controls participated in this study. The patient group consisted of more males (40% females) than the control group (67% females) (*χ* ^2^(1) = 7.702, *p* = 0.006). Patients (M = 72.9 years, SD = 9.5) were older (*t*(103) = 2.098, *p* = 0.038) than controls (M = 67.9 years, SD = 11.9).

#### 2.1.3. Education

Education was measured on a 5-point rating scale (1 = primary school, unfinished; 2 = primary school; 3 = secondary school; 4 = higher secondary school; 5 = higher vocational training or university degree). Level of education was matched between groups (Mann-Whitney  *U* = 1151.500, *Z* = −0.729, *p* = 0.466).

#### 2.1.4. Comorbidities

Comorbidities were recorded. As can be seen in Table 1, patients were significantly more often diagnosed with diabetes.

#### 2.1.5. Medical Ethical Committee

This study was approved by the medical ethical committee of the VU medical center. All participants received information about the study before signing an informed consent form.

## 3. Material and Procedure

### 3.1. Pain

Pain was assessed by visual analogue scales, a Faces Pain Scale, and a verbal descriptor scale.

Visual analogue scales: the* Colored Analogue Scale (CAS) *is a colored version of the visual analogue scale. To test pain intensity, the CAS Intensity was used. Here, subjects indicate how much pain they have on a scale ranging from “no pain” (light pink) to “maximal pain” (dark red). To test suffering from pain, the CAS Affect was used. The CAS Affect is a similar color-coded scale ranging from “no suffering” to “maximal suffering.” Both versions of the CAS range from 0 to 100.

The* Faces Pain Scale (FPS)* primarily measures pain intensity, but also pain affect [30]. The FPS consists of seven pictures of faces, with a pain expression ranging from “no pain” to “most severe pain.” Subjects choose a face that represents their pain best. Answer possibilities of the FPS range from 0 to 6.

Verbal descriptor scale: the* Number of Words Chosen-Affective (NWC-A)* is the affective part of the McGill Pain Questionnaire and was utilized to investigate the affective aspect of pain [31, 32]. Three adjectives, all expressing an increasing amount of pain, are read aloud by the examiner. Subjects have to indicate which of the three adjectives, if any, best represents their pain. Possible scores range from 0 to 15.

### 3.2. Cognition

#### 3.2.1. Memory

To assess memory, the following neuropsychological tests were administered.

The* Eight-Word Test* is part of the Amsterdam Dementia Screening test [33] and consists of three verbal memory subtests: immediate recall, delayed recall, and delayed recognition. In the* immediate recall subtest*, eight words are read aloud five times by the examiner and each time the participant is required to recall as many words from the list as possible. Maximum score for this subtest is 40. The* delayed recall subtest* is assessed after approximately 15 minutes. The participant is required to reproduce as many words from the original set of words as possible. Maximum score for this test is 8. The* delayed recognition subtest* is assessed immediately after the delayed recall test. The participant is required to recognize the initial set of eight words from a set of 16 words and indicate which words are new. Maximum for this test is 16.

*Face Recognition *is a subtest of the Rivermead Behavioral Memory Test (RBMT) and measures visual recognition memory [34]. Ten pictures of faces are presented to the participant in a fixed order. Approximately three minutes later the participant is asked to recognize the initial set of 10 faces from a set of 20 faces and indicate which faces are new. Maximum score for this test is 20.

*Picture Recognition *is another subtest of the RBMT and measures visual recognition memory. Twenty drawings of objects and animals are displayed to the participant in a fixed order. Approximately three minutes later the participant is asked to recognize the initial set of 20 drawings from a set of 40 drawings and indicate which drawings are new. For both RBMT subtests, number of correct answers minus number of incorrect answers was taken as an outcome measure. Maximum score for this test is 40.

The* Knox Cube Test* was used to assess visual attention and memory [35]. Subjects are asked to imitate the pattern tapped by the researcher on a wooden model of four small, aligned blocks. The length of the tapped pattern increases, until the subject is unable to reproduce it. The number of correct test trials was recorded as outcome measure. Maximum score for this test is 15.

#### 3.2.2. Executive Functioning

To assess executive functioning, the following neuropsychological tests were administered.

The* Digit Span Backward *test from the Wechsler Memory Test was used to assess working memory [36]. Subjects are instructed to repeat a string of verbally presented digits backwards. The total amount of correct responses was used as the outcome measure. Maximum score for this test is 21.

The* Key Search test* of the Behavioural Assessment of the Dysexecutive Syndrome (BADS) measures planning, monitoring, and regulation of behavior [34]. The subject is required to draw the route they would take in the hypothetical situation of trying to find a lost key on a large field. The drawn route is scored according to a predefined set of rules. This score was used as outcome measure. Maximum score for this test is 16.

The* Rule Shift test* of the BADS measures cognitive flexibility and attention [37]. A set of playing cards is shown to the subject. In the first part, a response pattern is established according to a simple rule (i.e., “Yes” for a red card, “No” for a black card). For the second part, subjects are required to respond differently to the same set of cards. The outcome measure was the number of correct answers. Maximum score for this test is 19.

*Category Fluency* measures verbal ability and executive control: the subject is required to name as many animals within 60 seconds and as many professions in another trial of 60 seconds [38]. Total number of mentioned instances served as outcome measure.

*The Picture Completion test* of the Groninger Intelligence Test (GIT) was used to assess visual perception and alertness to detail [39]. Subjects are asked to complete a partly drawn figure, each with increasing difficulty (i.e., less complete). Number of correct answers was taken as outcome measure. Maximum score for this test is 20.

#### 3.2.3. Attention

To assess attention, the* Digit Span Forward *test from the Wechsler Memory Test was administered [36]. Subjects are instructed to repeat a string of verbally presented digits. Number of digits per string was increased until the subject was unable to reproduce the string. The total amount of correct responses was used as the outcome measure. Maximum score for this test is 21.

### 3.3. Mood

Depressive symptoms were evaluated with the Beck Depression Inventory (BDI) [40]. The administered BDI consists of 20 questions, all addressing depressive symptoms, with answer options ranging from no symptoms (0) to severe symptoms (3).

Additionally, the depression and anxiety subscales of the* Symptom Check List-90* were administered [41]. The subtest Depression consists of 15 symptoms, with answer options ranging from 1 (“not bothered at all”) to 5 (“extremely bothered”); the subtest Anxiety addresses 10 symptoms with the same answer options.

### 3.4. Procedure

Subjects were recruited through outpatient clinics of hospitals, patient societies, and immediate surroundings. Patients and controls were assessed in their own home and were given the freedom to choose the day and time of assessment. By this, we hope to overcome the possibility of suboptimal performance. The order of neuropsychological tests, pain assessments, and mood questionnaires was as follows: MMSE, CAS, FPS and NWC-A, Eight-Word Test-immediate recall condition, BADS rule-shifting subtest, BADS key search subtest, Eight-Word test-long-term recall and recognition condition, Digit Span Forward, Digit Span Backward, RBMT Faces (present faces), Fluency-animals, RBMT Faces-recognition condition, RBMT Pictures-presentation, Fluency-professions, RBMT Pictures-recognition condition, KNOX cubes, figure completion test, BDI, and SCL90 subscales anxiety and depression. Approximate duration of assessment was 1.5–2 hours.

### 3.5. Statistical Analyses

Statistical Package for the Social Sciences (SPSS), version 21, and Lavaan package in R were utilized to perform statistical analyses [42]. In order to determine whether PD patients and controls differed on pain measures, an Analysis of Covariance (ANCOVA) was conducted with pain as dependent variable, group and gender as factors, and age as covariate. Confirmatory factor analysis (CFA) was performed to determine domains in cognitive scores. Four linear multiple regression analyses were performed within the PD group, all with one pain measure as outcome variable and memory, executive functioning, and attention performance and mood and gender as predictors. The alpha level was set at 0.05.

### 3.6. Cognitive Domains

A factor analysis was performed to reduce the number of predictors in the analysis. The 12 cognitive subtests were entered into a CFA analysis. Two domains were created: the domain Memory entailed three subtests of the Eight-Word Test (immediate recall, delayed recall, and recognition), RBMT Face and Picture Recognition, KNOX, and Picture Completion. The domain executive functioning was represented by BADS Rule Shift test, BADS Key Search test, Digit Span Backward, and Category Fluency. The Comparative Fit Index (CFI) of the CFA was 0.912, and the Root Mean Square Error of Approximation (RMSEA) was 0.097 (*p* = 0.009), indicating that domains were indeed measuring the same construct. Digit Span Forward (DSF) served as an individual variable representing attention.

## 4. Results

### 4.1. Differences in Cognitive Functioning between PD Patients and Controls

The average MMSE score for patients (M = 26.14, SD = 3.57) was significantly lower than for control subjects (M = 28.00, SD = 2.09, Mann-Whitney *U* = 896.000, *Z* = −3.076, *p* = 0.002). Patients performed worse on all neuropsychological subtests (see Table 2 for details).

### 4.2. Differences in Pain between PD Patients and Controls

After controlling for age, there was no main effect of group on CAS Intensity (*F*(1, 100) = 1.905; *p* = 0.171).

There was a significant main effect of group on CAS Affect (*F*(1, 100) = 6.803; *p* = 0.010, partial *η* ^2^ = 0.064). Additionally, the effect of gender as a factor showed a trend on CAS Affect, with women experiencing more pain then men (*F*(1, 100) = 3.433; *p* = 0.067, partial *η* ^2^ = 0.033), and there was no significant interaction between these variables (*F*(1, 100) = 0.170; *p* = 0.681, partial *η* ^2^ = 0.002).

Patients showed to experience more pain as measures on the FPS than controls after controlling for age (*F*(1, 100) = 6.191; *p* = 0.014, partial *η* ^2^ = 0.058). Here, the effect of gender as a factor showed a trend (*F*(1, 100) = 3.253; *p* = 0.074, partial *η* ^2^ = 0.032), with women scoring higher on the FPS but without a significant interaction between these variables (*F*(1, 100) = 2.143; *p* = 0.146, partial *η* ^2^ = 0.021).

There was a main effect of group on NWC-A (*F*(1, 100) = 15.503; *p* = 0.000, partial *η* ^2^ = 0.134), as well as an effect of gender as a factor (*F*(1, 100) = 7.264; *p* = 0.008, partial *η* ^2^ = 0.068). There was no significant interaction between group and gender (*F*(1, 100) = 0.030; *p* = 0.864, partial *η* ^2^ = 0.000). See Table 3 for scores on the pain scales.

### 4.3. Linear Regression Analyses to Assess the Relationship between Pain and Cognition

Four multiple linear regression analyses were calculated to predict pain (as measured by the four different pain measures) based on Memory, EF, attention, gender, and Mood for the group of PD patients.

No significant regression equation was found for the multiple linear regression to predict outcome on CAS Intensity (*F*(5, 36) = 1.355, *p* = 0.264), with an *R* ^2^ of 0.158.

The regression model for predicting scores on FPS significantly accounted for approximately 46% of the variance (*R* ^2^ = 0.463, *F*(5, 36) = 6.207, *p* < 0.001). However, none of the cognitive measures significantly contributed to the model. Mood and (male) gender showed a trend in predicting pain as measured by FPS.

The regression model for CAS Affect accounted for 25.7% of the variance (*F*(5) = 2.495, *p* = 0.049). As shown in Table 4, none of the cognitive measures contributed to the model. In this model, only mood significantly predicted scores on CAS Affect.

Similar results were found for the regression model predicting scores on the NWC-A (*R* ^2^ = 0.463, *F*(5) = 6.207, *p* < 0.001): none of the cognitive measures were contributing to the model. Mood was the only significant predictor in this model. See Table 4 for details.

## 5. Discussion

To our knowledge, this is the first study to address the association between cognition and pain in PD patients. Although pain intensity did not significantly differ between groups consistently (CAS Intensity, FPS), PD patients experienced more pain effect (CAS Affect, NWC-A).

When compared to controls, it has been found that PD patients experience pain more frequently [4]. However, this has not been found consistently: others (e.g., Quittenbaum and Grahn) report that pain in PD does not differ from pain in the general population [3]. Even though Quittenbaum and Grahn did not include an assessment of all aspects of pain, we propose that this inconsistency in findings might be explained by a difference between the aspects of pain: in our sample, patients may have* suffered* more from pain than controls, whereas the sensory-discriminative aspect was comparable to controls. This discrepancy might be explained by the progression of PD-neuropathology throughout the pain systems: the lateral pain system (sensory-discriminative aspect) remains relatively intact, whereas the medial pain system, involved in the affective aspect of pain, shows more neuropathology [15, 27].

The main goal of this study was to examine a possible relationship between pain and cognition in PD. We hypothesized that this relationship could be either positive or negative and that specifically a negative relationship would be clinically relevant, considering the risk of undertreating pain in cognitively impaired patients who might have more difficulty communicating their pain to a physician. In contrast to our hypothesis, cognitive functioning showed no relationship with pain in our sample of PD patients. Yet similar to other studies, symptoms of mood disorders strongly influenced pain experience. In healthy elderly, a positive relationship between MMSE scores and pain tolerance has been found previously [26]. The relationship between everyday* clinical* pain and cognitive performance has been researched before, albeit not in Parkinson's disease: for a group of Alzheimer's disease patients, a positive correlation between executive functioning and pain was found. However, the authors emphasized the need to control for symptoms of mood disorders when studying the relation between pain and cognition [43]. This was confirmed in a study looking into patients with rheumatoid arthritis, where cognition was negatively correlated to pain [44]. This correlation disappeared when the authors additionally assessed the mediating effect of depression, indicating the major influence of depression on both pain and cognition.

One possible explanation for the lack of a significant association between pain and cognitive functioning might be that neural functional reorganization takes place to compensate for deficits on cognitive performance. These compensatory processes may result in the activation of other brain regions during tasks that measure memory and executive functioning than in controls. These brain regions may not be as involved in the processing of pain as they are in the controls. An example of this shift in recruited brain regions is found in multiple sclerosis (MS). In a study investigating MS patients, increased and additional activation of frontal and posterior parietal cortices was observed during several attention tasks. These areas were not or only partly activated in controls, during the same tasks [45, 46]. In PD, a recent study investigated functional connectivity and cognitive functioning in PD patients [47]. The authors concluded that, in the beginning of the disease, a state of* hyperconnectivity* arises and that a* hypoconnectivity* is associated with more cognitively impaired patients. They also state that this might be a compensatory mechanism of the brain. A similar mechanism might apply for pain: a decrease in gray matter in pain areas (e.g., ACC, insula) has been found for several chronic pain disorders and could even be the consequence of chronic pain [48]. A deviant neural functioning as a consequence of the disease (the compensatory neural mechanism) or of the symptoms themselves (pain) might therefore explain the lack of association between pain and cognition.

Our study confirms that mood disorders are the main contributor to pain experience in PD: symptoms of anxiety and depression were predictive for three out of four pain parameters. This finding is in line with previous research [49]. The relation between pain and mood could be bidirectional: symptoms of depression or anxiety might increase pain experience, and, vice versa, pain might exacerbate symptoms of depression and anxiety [49]. In our sample, NWC-A and CAS Affect showed the strongest relationship with mood. The FPS and CAS Intensity, both focusing on intensity (sensory-discriminative aspect) of pain, showed a weaker association [50]. This is an interesting finding, since it strengthens our belief that pain experience consists of several components, all of which should be paid equal attention. Clinically, it also indicates that pain treatment will probably benefit when mood is taken into account.

### 5.1. Limitations

Several limitations apply to this study. Firstly, regarding cognitive functioning, no patients showed severe cognitive impairment, which reduces the variability in the sample. Subsequently, our hypothesized relationship might not be as pronounced as it would have been when this increased variability would be present in our sample. We chose to exclude patients with a severe cognitive impairment because they are no longer able to fully understand the neuropsychological tests. However, this unfortunately results in a reduction of variability of cognitive functioning. Secondly, use of pain medication was not recorded. Analgesic medication (evidently) influences pain experience and therefore might also influence the relationship between cognition and pain. In addition, participants were not required to withhold dopaminergic medication. This might influence our results in two ways. Indirectly, the reduction of (painful) motor symptoms during the “on” period might bring pain relief. Directly, dopaminergic medication has an ameliorating influence on pain experience as it has been shown that hypofunctioning of nigrostriatal dopamine is associated with an increase in pain experience in healthy persons [51]. Indeed, PD patients show a lowered pain threshold during their “off” period, and the pain threshold returns to a normal level after levodopa administration [52], although this effect has also been found to differ depending on how the patient responds to dopaminergic medication [53]. The dopaminergic antinociceptive effect might therefore also have an influence on the association between pain and cognition in our study. Finally, several clinical and demographical variables have not been taken into account, such as disease duration and severity of the disease. These have been shown to influence pain experience [54]. Regarding the comorbidities, there were more patients with diabetes as comorbidity than controls, which poses the possibility of, for example, encephalopathy.

## 6. Conclusion

This study suggests that symptoms of mood disorders are an important predictor for the experience of pain in PD patients, probably of more significance than cognitive dysfunction in these patients, since we did not find a significant relationship between pain experience and cognition.

## Figures and Tables

**Table 1:** Comorbidities for both groups; ^*∗*^ *p* < 0.05.

Comorbidity	Controls	Parkinson's disease	Difference between groups	
	Percentage (number)	Percentage (number)	Chi-square	*p* value
Cardiac failure	28.1% (16)	29.2% (14)	0.015	0.901
Kidney disease	1.8% (1)	0% (0)	0.850	0.356
Lung disease	10.5% (6)	4.2% (2)	1.497	0.221
Diabetes^*∗*^	5.3% (3)	18.8% (9)	4.682	0.030^*∗*^
Hypertension	29.8% (17)	27.1% (13)	0.096	0.757
Peripheral arterial disease	8.8% (5)	14.6% (7)	0.869	0.351
Arthritis	12.3% (7)	8.9% (4)	0.301	0.583

**Table 2:** Cognitive performance in PD patients and controls. SD: standard deviation; MMSE: Mini-Mental State Examination; BADS: Behavioral Assessment of the Dysexecutive Syndrome; RBMT: Rivermead Behavioral Memory Test; ^*∗*^ *p* < 0.05, and ^*∗∗*^ *p* < 0.001.

	Controls		Parkinson's disease		Mann-Whitney *U* test (*Z*)	*p* value
	Mean (SD)	Range	Mean (SD)	Range		
MMSE	28.00 (2.09)	21–30	26.14 (3.57)	16–30	−3.076	0.002^*∗*^
Eight-Word Test: immediate recall subtest	32.23 (5.00)	20–40	25.86 (7.94)	4–38	−4.542	<0.001^*∗∗*^
Eight-Word Test: delayed recall subtest	5.81 (1.55)	1–8	4.16 (2.37)	0–8	−3.804	0.001^*∗*^
Eight-Word Test: delayed recognition subtest	15.37 (0.86)	12–16	14.14 (2.30)	6–16	−3.398	0.001^*∗*^
Rule Shift test (BADS)	17.16 (3.05)	9–19	14.75 (4.17)	6–19	−3.311	0.001^*∗*^
Key Search test (BADS)	10.61 (4.07)	3–16	8.55 (4.33)	2–16	−2.892	0.004^*∗*^
Digit Span Forward	13.28 (2.96)	8–21	11.84 (2.96)	5–19	−2.496	0.013^*∗*^
Digit Span Backward	9.00 (3.05)	3–18	7.34 (2.69)	3–14	−3.018	0.003^*∗*^
Face Recognition (RBMT)	16.88 (3.14)	6–20	14.41 (4.50)	2–20	−3.119	0.002^*∗*^
Picture Recognition (RBMT)	38.58 (2.57)	26–40	36.55 (5.90)	12–40	−2.055	0.040^*∗*^
Category Fluency	39.30 (11.85)	6–67	30.48 (13.05)	5–70	−4.107	<0.001^*∗∗*^
Knox Cube Test	11.14 (1.70)	7–15	10.14 (1.91)	5–14	−2.883	0.004^*∗*^
Picture Completion test (GIT)	11.51 (3.36)	5–18	9.75 (3.98)	2–19	−2.635	0.008^*∗*^

**Table 3:** Pain scores in PD patients and controls. SD: standard deviation, ^*∗*^ *p* < 0.05, and ^*∗∗*^ *p* < 0.001.

	PD patients		Controls		Main effect of group	
	Mean (SD)	Range	Mean (SD)	Range	*F*-value (df)	*p* value (partial *η* ^2^)
Colored Analogue Scale (CAS) Intensity	25.74 (25.45)	0–95	30.88 (27.13)	0–92	1.905 (1, 100)	0.171 (0.019)
Faces Pain Scale (FPS)	1.49 (1.31)	0–5	2.17 (1.81)	0–7	6.191 (1, 100)	0.014 (0.058)^*∗*^
Colored Analogue Scale (CAS) Affect	20.16 (24.95)	0–75	32.04 (27.27)	0–90	6.803 (1, 100)	0.010 (0.064)^*∗*^
Number of Words Chosen-Affective (NWC-A)	2.02 (2.66)	0–10	4.13 (3.64)	0–13	15.503 (1, 100)	0.000 (0.134)^*∗∗*^

**Table 4:** Four multiple linear regression analyses were performed, each with a different pain measure as predicted variable. Coding for “Gender”: 1: female, 2: male. *B*: unstandardized beta; SE_*B*_: standard error of the *B*; *β*: standardized beta; ^*∗*^ *p* < 0.10, ^*∗∗*^ *p* < 0.05, and ^*∗∗∗*^ *p* < 0.001.

	(1) Colored Analogue Scale (CAS) Intensity			(2) Faces Pain Scale (FPS)			(3) Colored Analogue Scale (CAS) Affect			(4) Number of Word Chosen-Affective (NWC-A)		
	*B*	SE_*B*_	*β*	*B*	SE_*B*_	*β*	*B*	SE_*B*_	*β*	*B*	SE_*B*_	*β*
Memory	5.936	7.101	0.186	−0.643	0.411	−0.316	−3.860	6.608	−0.122	−0.968	0.761	−0.226
Executive functioning	−5.773	8.154	−0.165	0.348	0.472	0.156	3.607	7.588	0.106	0.455	0.874	0.097
Mood	10.050	5.099	0.344	0.529	0.295	0.284	13.084^*∗∗*^	4.746^*∗∗*^	0.451^*∗∗*^	2.198^*∗∗∗*^	0.547^*∗∗∗*^	0.560^*∗∗∗*^
Attention	−0.422	4.963	−0.016	0.134	0.288	0.078	0.088	4.619	0.003	0.296	0.532	0.082
Gender	−4.692	8.475	−0.089	−0.997^*∗*^	0.491^*∗*^	−0.298^*∗*^	−5.230	0.888	−0.101	−1.103	0909	−0.157
